# Response to Importation of a Case of Ebola Virus Disease — Ohio, October 2014

**Published:** 2014-11-21

**Authors:** Carolyn L. McCarty, Colin Basler, Mateusz Karwowski, Marguerite Erme, Gene Nixon, Chris Kippes, Terry Allan, Toinette Parrilla, Mary DiOrio, Sietske de Fijter, Nimalie D. Stone, David A. Yost, Susan A. Lippold, Joanna J. Regan, Margaret A. Honein, Barbara Knust, Christopher Braden

**Affiliations:** 1 Epidemic Intelligence Service, CDC; 2 Ohio Department of Health; 3 CDC Ebola Response Team, Ohio; 4 Summit County Public Health; 5 Cuyahoga County Board of Health; 6 Cleveland City Health Department; 7 CDC Ebola Outbreak Response Global Migration Task Force

On September 30, 2014, the Texas Department of State Health Services reported a case of Ebola virus disease (Ebola) diagnosed in Dallas, Texas, and confirmed by CDC, the first case of Ebola diagnosed in the United States (1). The patient (patient 1) had traveled from Liberia, a country which, along with Sierra Leone and Guinea, is currently experiencing the largest recorded Ebola outbreak (2). A nurse (patient 2) who provided hospital bedside care to patient 1 in Texas visited an emergency department (ED) with fever and was diagnosed with laboratory-confirmed Ebola on October 11 (1), and a second nurse (patient 3) who also provided hospital bedside care visited an ED with fever and rash on October 14 and was diagnosed with laboratory-confirmed Ebola on October 15. Patient 3 visited Ohio during October 10–13, traveling by commercial airline between Dallas, Texas, and Cleveland, Ohio (Figure). Based on the medical history and clinical and laboratory findings on October 14, the date of illness onset was uncertain; therefore, CDC, in collaboration with state and local partners, included the period October 10–13 as being part of the potentially infectious period, out of an abundance of caution to ensure all potential contacts were monitored. On October 15, the Ohio Department of Health requested CDC assistance to identify and monitor contacts of patient 3, assess the risk for disease transmission, provide infection control recommendations, and assess and guide regional health care system preparedness. The description of this contact investigation and hospital assessment is provided to help other states in planning for similar events.

The movements and activities of patient 3 were identified and confirmed through interviews with the patient and close contacts, social media, press releases, and an airport/airline investigation. During her time in Ohio, patient 3 had contact with two household members (one of whom was interviewed and monitored in Texas after traveling there and is not included in the number monitored in Ohio) as well as contact with 10 friends and family members and 60 patrons and employees at one store. Seventeen airline and airport personnel and 76 airline passengers also were monitored in Ohio because of contact with patient 3. Some exposures were brief, whereas others lasted several hours; some likely included direct skin-to-skin exposure. Contacts were interviewed to determine risk for exposure and monitored by local health jurisdictions in Ohio, with the majority of contacts residing in metropolitan areas near Cleveland and Akron. All 164 Ohio contacts were asked to monitor their temperature and symptoms twice a day for the 21-day incubation period. Based on Ohio’s risk stratification, which was similar but slightly more restrictive than CDC’s *Interim U.S. Guidance for Monitoring and Movement of Persons with Potential Ebola Virus Exposure* (3), 50 contacts who had no direct contact and were not within a 3-foot (1-meter) radius of the patient but were in the same enclosed space for less than an hour self-monitored only; 94 contacts who similarly had no direct contact and were not within a 3-foot radius but had a more prolonged period in the same enclosed space with the patient self-monitored and reported the results once daily to local public health officials; 20 contacts who were within a 3-foot radius of the patient and in the same enclosed space for 1 hour or more were directly actively monitored through twice-daily check-ins from local public health officials (once in person and once by phone). All 20 contacts under direct active monitoring had movement restrictions, including three who were under home quarantine because they reported household or likely direct skin-to-skin contact with the patient. Contacts were, in general, cooperative with monitoring, but there were extensive efforts required to ensure continuity of monitoring because many contacts were identified as a contact in one health jurisdiction (e.g., airport location) and had to be transferred to another health jurisdiction for daily monitoring based on their residence. As of November 3, the end of the 21-day incubation period and the final day of monitoring, no additional Ebola-infected patients had been identified, and none of the 164 monitored contacts had reported Ebola symptoms that resulted in testing.

Onsite technical consultations were conducted rapidly with seven hospital systems across the northeast Ohio region, identified by local health jurisdictions, to assess preparedness to care for a contact who developed symptoms of Ebola. All seven hospitals were determined to have capacity for isolation and transfer of a patient with suspected Ebola; five were deemed fully capable of providing care during the 72-hour Ebola evaluation period.* During the response, local health jurisdictions developed plans to coordinate emergency medical services transport of a patient who developed Ebola-like symptoms to minimize exposure of first responders and to direct the patients to appropriate facilities with personnel who were trained and prepared to accept these patients. Recognition of factors that could limit a hospital’s ability to provide Ebola patient care has prompted discussions about implementing a regional collaboration among health care systems to enable resource sharing to extend capacity.

This response required substantial time, resources, and coordination between local health jurisdictions in 19 Ohio counties, the state health department, federal public health authorities, and the regional health care system. This response highlighted the need for specific plans to be developed in advance for various potential situations, including identification of screening facilities for the triage of persons under investigation if the designated Ebola treatment facility reaches capacity†; identification of emergency medical services to transport persons under investigation safely to the nearest screening or treatment facility; identification and monitoring of large numbers of contacts; and following up on difficult-to-reach contacts to ensure their symptoms are monitored daily. Future responses could benefit from sharing of best practices from Ohio’s response, such as working with the state or local health department to mobilize staff to monitor large numbers of contacts and the daily posting of the number of contacts being monitored in each risk stratification category on the Ohio Department of Health website to facilitate communication with the public during a time of high public anxiety.

## Figures and Tables

**FIGURE:**
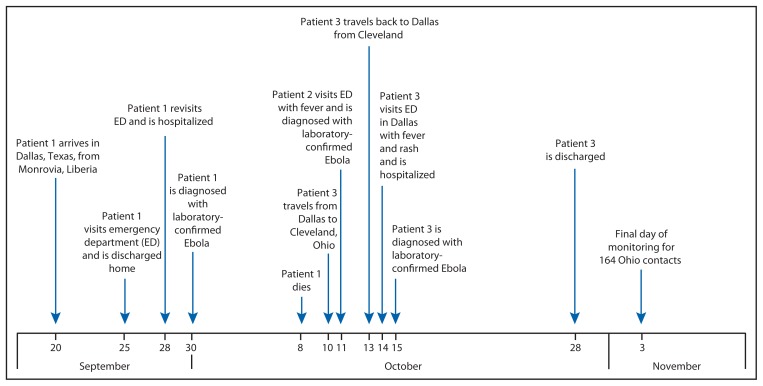
Timeline of events relevant to diagnosis of Ebola virus disease (Ebola) in patient 3 — Ohio and Texas, September 20–November 3, 2014

## Footnotes

* Additional information available at http://www.cdc.gov/vhf/ebola/hcp/considerations-discharging-pui.html.

† Additional information available at http://www.cdc.gov/vhf/ebola/hcp/case-definition.html.
